# Evaluation of the Vapor Hydrolysis of Lithium Aluminum Hydride for Mobile Fuel Cell Applications

**DOI:** 10.1021/acsaem.2c00891

**Published:** 2022-07-01

**Authors:** Elizabeth. Ashton, William. C. Oakley, Paul Brack, Sandra E. Dann

**Affiliations:** Department of Chemistry, School of Science, Loughborough University, Loughborough LE11 3TU, U.K.

**Keywords:** vapor hydrolysis, LiAlH_4_, hydrogen production, carbon dioxide, water retention

## Abstract

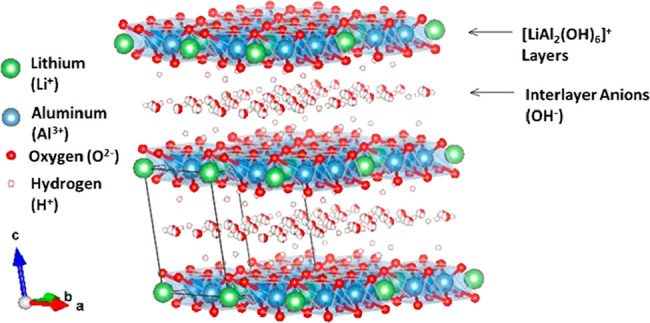

The controlled vapor hydrolysis of LiAlH_4_ has been investigated as a safe and predictable method to generate hydrogen for mobile fuel cell applications. A purpose-built vapor hydrolysis cell manufactured by Intelligent Energy Ltd. was used as the reaction vessel. Vapor was created by using saturated salt solutions to generate humidity in the range of 46–96% RH. The hydrolysis products were analyzed by thermogravimetric analysis (TGA) and powder X-ray diffraction and compared with possible hydroxide-based phases characterized using the same methods. Analysis of the products of the LiAlH_4_ vapor hydrolysis reaction at a relative humidity in excess of 56% indicated complete decomposition of the LiAlH_4_ phase and formation of the hydrated layered double hydroxide, [LiAl_2_(OH)_6_]_2_CO_3_·3H_2_O, rather than the simple salts, LiOH and Al(OH)_3_, previously suggested by the literature. The high level of hydration of the layered double hydroxide (LDH) (12% wt water) and the presence of carbonate indicated that the feed stream was contaminated with CO_2_ and that the highly hydrated and hygroscopic product would be detrimental to the mobile hydrogen production process, restricting recyclability of the water fuel cell byproduct and lowering the gravimetric density of LiAlH_4_. Carrying out the vapor hydrolysis reaction in a glovebox in the absence of CO_2_ indicated that the hydroxide derivative of the LDH, [LiAl_2_(OH)_6_]OH·2H_2_O, could be formed instead, but the water content was even more significant, equating to 17% of the carried weight. TGA showed that water was retained up to 300 and 320 °C in the two phases, making thermal recycling of the water retained impractical and casting doubt on whether generating hydrogen on the move by vapor hydrolysis of LiAlH_4_ is practical.

## Introduction

Road transport is one of the largest contributors to climate change due to emissions of greenhouse gases. Therefore, in line with the Paris agreement, many countries have announced their intention to cease petrol and diesel vehicle production after 2040 to reduce reliance on fossil fuels and curb emissions.^1−3^ In order for this target to be met, the production and sales of zero-emission vehicles must increase.

One of the most promising methods of electricity generation for automobiles from renewable resources is via the use of proton exchange membrane hydrogen fuel cells. Hydrogen fuel cells are an efficient method of producing clean electricity, producing only water as a waste product if the hydrogen intake is free of contaminants. Difficulty however arises with the storage and delivery of hydrogen.

Hydrogen gas has high diffusivity and a low liquefaction temperature (24 K) and is extremely flammable. Therefore, storage and delivery systems must incorporate various safety designs to contain hydrogen efficiently and prevent leakage and ignition. Present methods use high-pressure gas cylinders to store hydrogen gas; however, the large weight and volume of these vessels limit their practicality. Current automobile models accommodate two to four large highly pressurized cylinders to ensure enough hydrogen gas to fuel a competitive driving range to gasoline-fueled vehicles (502 km, Toyota Mirai).^4^ Hydrogen gas can be refueled easily via a similar method to the traditional refueling of an automobile with gasoline. Hydrogen gas refueling takes only a minute or two, while recharging a battery can take anywhere between 30 min to 12 h, depending on the size of the battery and the charge point speed. For example, the Tesla Model S Long Range (2019) charge time is 15 h at a charge speed of 7 kW to achieve empty to full charge for a 100 kW h battery. Rapid chargers (150 kW) can be used to achieve a charge time of 1 h for a 300 mile driving range.^5^

However, due to the high pressures of the cylinders and high flammability of the hydrogen gas, vigorous safety testing is required to ensure that the cylinders and automobile models meet international safety standards. Increasing the ease of use and safety of hydrogen storage and delivery is therefore at the forefront of recent research for alternative sources of hydrogen fuel.

The U.S. Department of Energy (DOE) has set certain targets for on-board hydrogen storage for light duty vehicles.^6^ These targets aim to achieve high volumetric and gravimetric energy densities (1.7 kW h/L and 2.2 kW h/kg, respectively), while ensuring that safety and performance requirements are met. Devices should be operable at near ambient temperatures (min/max delivery −40/85 °C) and pressure (min/max delivery 5–2 bar) and surpass the energy density of already well-established energy delivery technologies; such as the lithium-ion battery with a volumetric energy density of 0.5 kW h/L and a gravimetry energy density of 0.2 kW h/kg.^7^ Additionally, the cost of the fuel itself must be comparable to that of gasoline and lithium-ion batteries if it is to have an impact on the automotive industry.

Hydrolysis of light metal hydrides such as NaBH_4_, LiBH_4_, and LiAlH_4_ offer a means of hydrogen generation at ambient temperature and pressures. Emphasis has been toward NaBH_4_ due its high gravimetric and volumetric energy densities; however, problems arise with the insolubility of the solid byproduct (NaBO_2_·*x*H_2_O), fouling the reaction and preventing the reaction from achieving completion. The solubility of NaBO_2_ is only 28 g per 100 g water; therefore, large amounts of water are required to keep the byproduct in solution, decreasing the overall gravimetric density of the system.^8^ As stated by Huang et al., the concentration of NaBH_4_ must be kept below 20 wt % to keep the byproduct in solution.^9^ Therefore, according to eq 1, the theoretical 10.8 wt % hydrogen is reduced to only 4 wt % hydrogen (when *x* ≈ 8.5), well below the minimum density required by the DOE for mobile applications
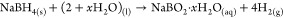
1

To overcome issues with solubility and crystallization of byproducts, research has shifted to reactions involving solid NaBH_4_. Catalysts and acids that favor the production of hydrogen are added to a water feed or added as a solid to the NaBH_4_ before the reaction with liquid water. Addition of catalysts however decreases the volumetric and gravimetric energy densities of the system.^10^

Research carried out by Marreroalfonso et al. has explored vapor hydrolysis as a method of hydrogen production from NaBH_4_, where steam is passed over a solid sample of NaBH_4_ in a reaction vessel.^10^ No catalysts or acidic conditions are required to achieve yields of 90% of the theoretical maximum hydrogen production at 110 °C. The reaction kinetics of NaBH_4_ with water vapor are faster than that with aqueous water. The hydroscopic byproduct NaBO_2_·*x*H_2_O is however still produced in varying degrees of hydration.^10^ In order for practical mobile system design, it is paramount to reduce the amount of hydration to increase the gravimetric density (in line with DOE requirements). At the same time, it would be advantageous if the water formed as a byproduct from hydrogen production could be recycled for the vapor hydrolysis of the hydride fuel rather than needing to carry additional water for this purpose.

When performing vapor hydrolysis of NaBH_4_, Matthews et al. also noticed that at elevated temperatures (>110 °C), problems still arose with the formation of the sodium borate byproduct.^11^ An insoluble shell of the byproduct was shown to form around the solid NaBH_4_ pellet as it reacted with the water vapor. As a result, the reaction rate decreased due the thickness of the layer increasing and preventing water vapor from penetrating into the remaining material.^11^ The reaction did not achieve completion, and typically, 10% of the material was left untouched by the reaction, irrespective of the relative humidity (RH) used. To increase ease of handling of the reactive complex hydrides during hydrolysis reactions, the powdered material is normally pressed into pellets; however, due to the insoluble byproduct formation in the case of NaBH_4_, the density of pellets must be low to produce good porosity and penetration of the vapor and ensure that the maximum hydrogen gas can be released. These issues highlight further challenges with using NaBH_4_ as a hydrogen fuel.

As an alternative, alkali-metal aluminum hydrides also have the potential to provide clean hydrogen for fuel cell use. LiAlH_4_ offers similar gravimetric (3.5 kW h/kg) and volumetric (3.5 kW h/L) energy densities to NaBH_4_; gravimetric and volumetric energy densities are 4.1 kW h/kg and 4.4 kW h/L, respectively. The hydrolysis reaction of LiAlH_4_ (eq 2) is however highly exothermic with the potential to ignite the hydrogen during production.^12^ Little attention has therefore been focused on these materials due to safety concerns.
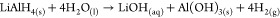
2

To date, research on this system has focused more on yields of hydrogen production and reaction parameters to increase the yield, with little attention toward materials characterization of the reaction product, as determined in detail for NaBH_4_. For this reason, it is uncertain what affects the reaction byproducts formed and how they may impact water retention plus overall gravimetric and volumetric energy densities of the whole system.

In this study, we present a method of controlled vapor hydrolysis of lithium aluminum hydride using a purpose-built system to overcome the issues with safety and extensive characterization of the products formed. The predicted reaction products [LiOH and Al(OH)_3_] presented in eq 2 are very hydroscopic, theoretically decreasing the overall gravimetric density of the system by retaining water and decreasing recyclability of the water in the closed system.^12^ Primarily, the focus is on the characterization of the reaction products to determine whether or not the predicted simple salt hygroscopic products are actually what is produced. Ways to reduce the water uptake, increase the water recyclability, and optimize the system have also been investigated.

## Experimental Section

All materials used are listed in Table 1, along with the supplier and purity.

**Table 1:** List of Chemicals Used for the Hydrolysis of Lithium Aluminum Hydride Using RH

name	supplier	purity
potassium carbonate	Fisher Scientific	>99%
magnesium nitrate	Sigma-Aldrich	>99%
sodium chloride	Fisher Scientific	>99%
potassium chloride	VWR Chemicals	100%
potassium nitrate	Fisher Scientific	>99%
lithium aluminum hydride	Sigma-Aldrich	>99.95%
aluminum chloride hexahydrate	Sigma-Aldrich	>99.5%
lithium hydroxide monohydrate	Sigma-Aldrich	>99.995%
sodium carbonate	Fisher Scientific	>99%
aluminum hydroxide	Sigma-Aldrich	97%

### Vapor Hydrolysis of Lithium Aluminum Hydride

**Figure 1:**
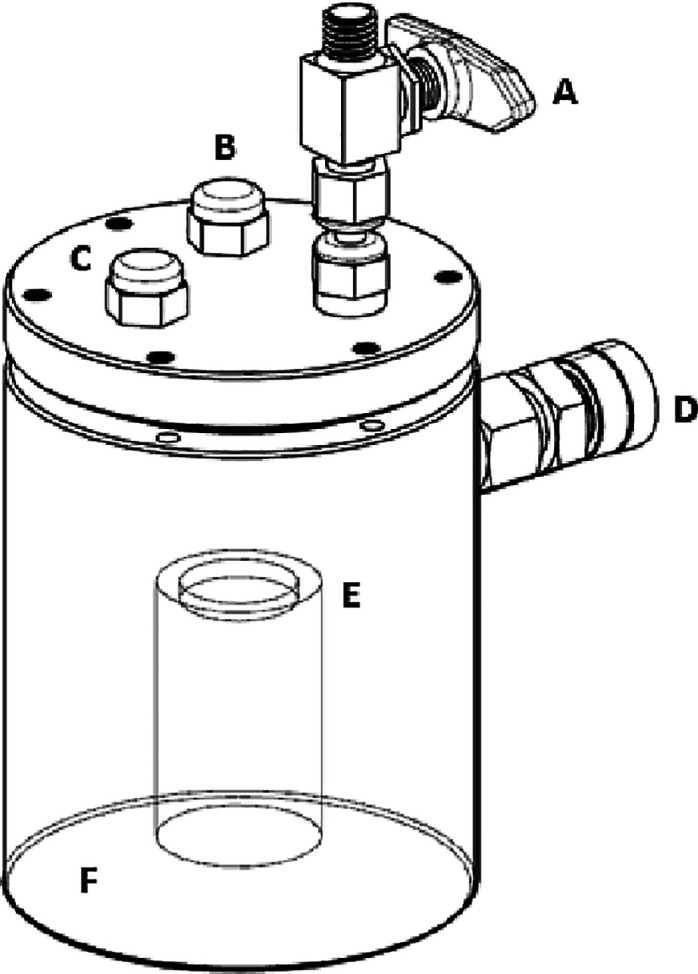
Schematic of the purpose-built vapor hydrolysis cell, designed by Paul Brack and engineered by Intelligent Energy Ltd: (a) Swagelok on–off valve for gas outlet, (b) port for the pressure/temperature sensor to be connected, (c) purge gas inlet, (d) pressure relief valve (3.5 bar), (e) pellet of complex hydride (e.g., LiAlH_4_), and (f) saturated salt solution to create humidity.

A schematic of the purpose-built vapor hydrolysis cell used is displayed in Figure 1. Different saturated salt solutions were used in the base of the cell to produce RH values of 46% (potassium carbonate), 56% (magnesium nitrate), 76% (sodium chloride), 86% (potassium chloride) and 96% (potassium nitrate), respectively.

The saturated salt solution maintains an equilibrium with the amount of water in the solution and the water vapor in the air at a constant % RH, which can be maintained even when small amounts of water are added or removed from the system. RH is the ratio (Equation 3) of the partial pressure of water within a mixture (*p*H_2_O) to the partial pressure of water vapor at the equilibrium (p*H_2_O) over a pure water surface.
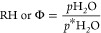
3

The RH above a pure water solution is 100%. Saturated salt solutions generate an RH of less than 100% depending on which salt is used and the temperature.

The minimum amount of saturated salt solution required to achieve the desired RH was calculated using the method stated by Timar-Balazsy and Eastop.^13^ This allowed the calculation of the surface area of the solution required for a specific volume of a closed humidifier chamber. The volume of the chamber was calculated to be 405 cm^3^; therefore, a surface area of at least 27.36 cm^2^ was required to achieve the desired humidity (∼30 mL).

Once the saturated solution was added, the vapor hydrolysis cell was closed and left to reach humidity equilibrium, which occurred after only 10 min. The humidity was recorded using a humidity probe (VAISALA humidity and temperature indicator HMI31 with an HMP35 probe).

Lithium aluminum hydride powder (0.007 mol) was placed on the platform in the middle of the cell, avoiding contact with the saturated solution below. The cell was closed to allow vapor hydrolysis to occur over a period of 24 h, then the sample was removed for characterization. The vapor hydrolysis reaction was performed in air and then again in a glovebox, under a nitrogen atmosphere, to exclude any CO_2_. The LiAlH_4_ was only packed into the VHC using a glovebox for the reactions performed with the exclusion of CO_2_. Characterization results of the products were compared to that of a mixture of the expected products, created by mixing a 1:1 of LiOH and Al(OH)_3_ using a mortar and pestle.

### Synthesis of [LiAl_2_(OH)_6_]_2_CO_3_

A pre-established synthesis by Chisem and Jones for [LiAl_2_(OH)_6_]_2_CO_3_, a layered double hydroxide (LDH), was followed to allow comparison to the vapor hydrolysis reaction product.^14^ Aluminum chloride hexahydrate solution (0.005 mol, 15 mL) was added dropwise to a solution (30 mL) of lithium hydroxide monohydrate (0.045 mol) and sodium carbonate (0.0024 mol) with vigorous stirring. The solution was heated for 18 h at 65 °C to ensure the completion of the reaction. The white precipitate product was collected by centrifugation and air-dried for 48 h.

### Synthesis of [LiAl_2_(OH)_6_]OH

To synthesize [LiAl_2_(OH)_6_]OH (LDH-OH), the method reported by Qu et al. was closely followed.^15^ 2 g of a 1:2 molar ratio of LiOH/Al(OH)_3_ mixture was ground together using a ball mill (500 rpm for 1 h) in the presence of water (3 mol). The products were collected and air-dried for 48 h before characterization.

### Materials Characterization

Powder X-ray diffraction (PXRD) was carried out on the samples collected. They were characterized using a Bruker D8 Advance powder diffractometer operating in the reflection geometry, with Cu Kα1 radiation and a LinkEye detector, calibrated against a silicon powder standard. Data were collected over the 2θ range of 5–80° with a count time of 15 s per 0.02 2θ step over a total run time of 15 h. The samples were rotated at 30 complete rotations per minute. XRD samples were prepared by grinding into a homogeneous powder and placed in an air-sensitive sample holder. The obtained X-ray diffraction (XRD) patterns were processed using the Bruker DIFFRAC.SUIT EVA (release 2011, version 2.1) to allow comparison of the diffraction data collated with the International Centre for Diffraction Data (ICDD) via phase matching algorithms.

Fourier transform infrared (FTIR) spectrometry was performed on all samples obtained using the PerkinElmer Spectrum 100 FTIR spectrometer with cesium iodide optics. Cesium iodide was used as an alternative to the usual potassium bromide as KBr is known to exchange with complex hydroxides and generate spurious bands. Absorption spectra were measured between 4000 and 450 cm^–1^. All samples were prepared by grinding a small amount of the sample (10 mg) with cesium iodide (50 mg) into a fine homogeneous powder and pressing into a thin, transparent pellet. The pressure of approximately 10 ton was applied for at least a minute. The IR data produced from the pellets were analyzed using PerkinElmer Spectrum (version 10.00.00.0018) and Dr. Friedrich Menges’s SpectraGryph (version 1.2.8) software.

Thermogravimetric analysis (TGA) and differential thermal analysis (DTA) were performed using a TA SDT Q600 instrument. Two alumina crucibles with a small amount of the homogeneous sample (5–10 mg) and an almost equal (±0.01 mg) amount of the alumina reference powder (Al_2_O_3_) were used for the analysis. The temperature was increased at a rate of 10 °C/min from 25 to 1000 °C. Data were analyzed using TA Instruments Universal Analysis 2000 (version 4.5A, build 4.5.0.5) software. The error of the TGA/DTA was ±0.0005 g.

## Results

### Vapor Hydrolysis of Lithium Aluminum Hydride at Different Humidities

**Figure 2:**
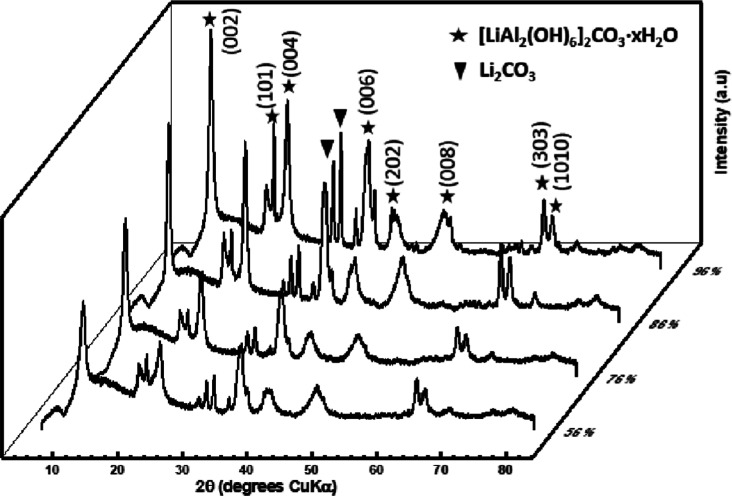
XRD data of LiAlH_4_ vapor hydrolysis products at varying RH values; 56% (magnesium nitrate), 76% (sodium chloride), 86% (potassium chloride), and 96% (potassium nitrate).

The reaction of LiAlH_4_ in the vapor hydrolysis cell at a humidity of 46%, generated by a saturated salt solution of potassium carbonate, did not achieve completion. The unreacted gray LiAlH_4_ powder was still present after the 24 h reaction time period. For this reason, no further analysis was conducted on the sample due to safety concerns of the unreacted LiAlH_4_. Figure 2 displays the XRD data obtained from the other four reactions, performed at 56, 76, 86, and 96% humidity, that were observed to reach completion via the formation of a white powder.

As the humidity was increased, the crystallinity of the product was observed to improve with more intense reflections of a narrower width at the half maximum height. 76% humidity was chosen to be used for following reactions due to the lower safety hazard of sodium chloride solutions compared to the oxidizing properties of the other saturated salts. Additionally, NaCl is of low cost and is very commercially available. This saturated salt also resulted in a low humidity requirement for the reaction to reach completion, therefore decreasing risks associated with the highly exothermic reaction of LiAlH_4_ with moisture.

**Figure 3:**
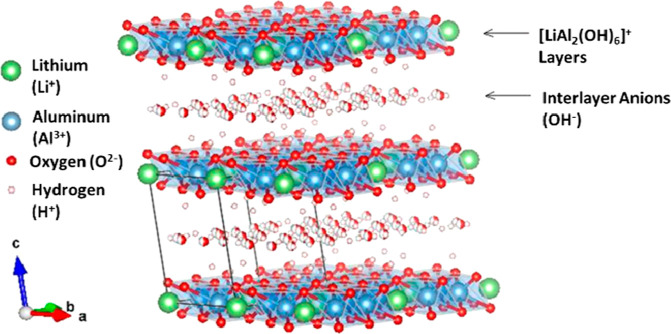
Lithium aluminum LDH ([LiAl_2_(OH)_6_]OH) structure using Rietveld refinement coordinates published by Thiel and Poeppelmeier.^18^

Instead of the predicted products, LiOH and Al(OH)_3,_ the XRD patterns produced matched the phase [LiAl_2_(OH_6_)]_2_CO_3_·*n*H_2_O (ICDD pattern 42-0729), which crystallizes with an LDH structure, with the reflection positions indicated by the star markers shown in Figure 2.^16,17^ LDHs are anionic clay materials that are composed of positively charged layers, neutralized by hydrated interlayer anions. The general structure of a layered hydroxide is given in Figure 3.

Divalent or trivalent metal cations form octahedral metal hydride sheets, with anions and water molecules filling the interlayer space. Lithium aluminum LDHs form when lithium ions fill the octahedral vacancies of Al(OH)_3_. The interlayer spacing is determined by the size of the anions that are filled in between the sheets.

**Figure 4:**
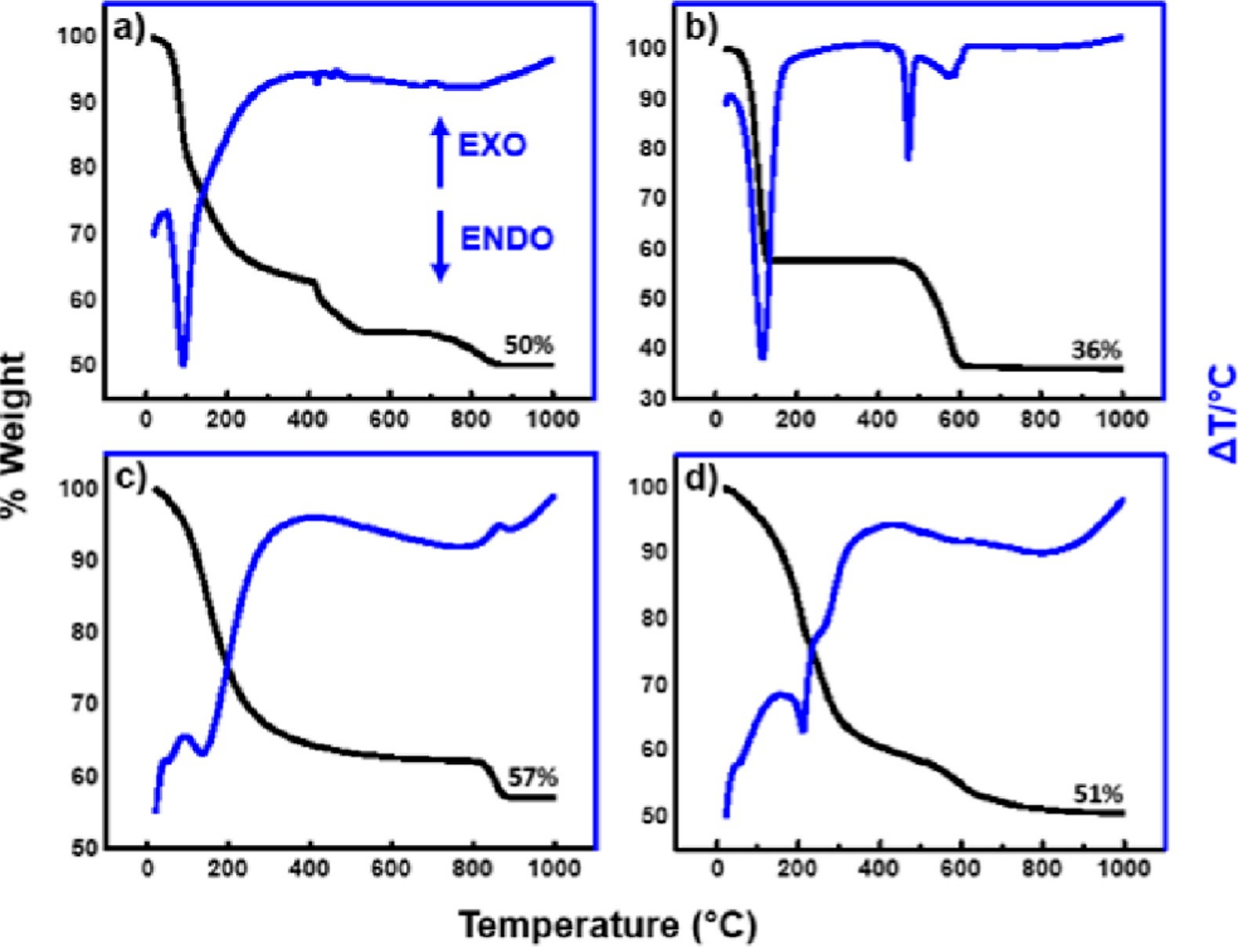
TGA (black) and DTA (blue) data of (a) a 1:1 mixture of LiOH/Al(OH)_3_, (b) LiOH, (c) Al(OH)_3_, and (d) LiAlH_4_ and the vapor hydrolysis product [LiAl_2_(OH)_6_]_2_CO_3_.

The well-known structure and characteristic thermal properties of LDHs mean that they can easily be identified using TGA and DTA (Figure 4d). Upon heating an LDH sample, it is expected to continuously lose weight due to the loss of physisorbed and intercalated water. First, physiosorbed water is lost at around 100 °C, which is also seen by an endothermic peak in DTA data.

Then, at higher temperatures (∼150–250 °C), water from the interlayer spaces is removed. As the temperature is increased further, dehydroxylation occurs, where hydroxyl ions from the [LiAl_2_(OH)_6_]^+^ sheets are removed, forming water and oxide anions (O^2–^). The fourth and final weight loss occurs usually at the highest temperatures and is due to the removal or decomposition of the anions in the interlayer space.^14,19,20^

Figure 4 compares the TGA and DTA data of the simple hydroxide products [LiOH and Al(OH)_3_] with those of the vapor hydrolysis product, showing a poor match.

The data for the previously predicted products (b) LiOH and (c) Al(OH)_3_ and a mixture of the two (a) differ from the thermogravimetric data for the vapor hydrolysis product (d). However, a good match is shown relative to that in the literature of the LDH-CO_3_ phase with three characteristic endothermic peaks and a continuous weight loss. The loss of weak physisorbed water is seen as the heating of the sample is begun. An endothermic peak at 213 °C indicated the loss of water from the interlayer spacing, followed by dehydroxylation and removal of the anions in the interlayer spaces, which occur between 230 and 350 °C, and a broad endothermic peak suggests that these last two decompositions have occurred at overlapping temperatures, as expected from the literature.^14,19,20^

The overall residual percentage weight from the TGA data of the expected products LiOH/Al(OH)_3_ was 50% in comparison to 51% for the vapor hydrolysis product. This suggests that in the presence of moisture and CO_2_, LiOH and Al(OH)_3_ can also react to form LDH-CO_3_ and Li_2_CO_3_. It is therefore unclear whether the simple salts [LiOH and Al(OH)_3_] are formed first and they then react to produce LDH-CO_3_ or the LDH-CO_3_ is formed directly from LiAlH_4_. XRD analysis of the TGA product of LDH-CO_3_ was also matched against the ICDD database, and it was determined that LiAlO_2_ was formed after heating. It can therefore be inferred from the mass loss of 49% in the TGA after heating the vapor hydrolysis product of LiAlH_4_ in air ([LiAl_2_(OH)_6_]_2_CO_3_·*x*H_2_O and Li_2_CO_3_) to 1000 °C that *x* = 3. The reactions that are predicted to occur during heating are summarized in Table 2.

**Table 2:** Reactions That Occur When Heating the Vapor Hydrolysis Reaction Products (LDH-CO_3_/Li_2_CO_3_) to 1000 °C During TGA

reaction equation	weight loss %
[LiAl_2_(OH)_6_]_2_CO_3_·3H_2_O + Li_2_CO_3_ → [LiAl_2_(OH)_6_]_2_CO_3_ + Li_2_CO_3_ + 3H_2_O	11
[LiAl_2_(OH)_6_]CO_3_ + Li_2_CO_3_ → 2LiAlO_2_ + Al_2_O_3_ + Li_2_CO_3_ + CO_2_ + 6H_2_O	29
2LiAlO_2_ + Al_2_O_3_ + Li_2_CO_3_ → 4LiAlO_2_ + CO_2_	9

The relative formula mass of [LiAl_2_(OH)_6_]_2_CO_3_·3H_2_O (LDH-CO_3_) is 439.95 g mol^–1^, resulting in 12% wt of water. Li_2_CO_3_ is not present after heating to 1000 °C during the TGA due to Li_2_CO_3_ reacting with Al_2_O_3_ to form LiAlO_2_. This reaction occurs between 800 and 900 °C and can be seen as a broad endothermic peak in the DTA curve.^21^

In addition to materials characterization, the TGA and DTA data also highlight that the LDH structure retains water to temperatures in excess of 300 °C. This is potentially problematic for on-board recyclability of water in a vapor hydrolysis fuel cell, where water recycling is needed to maintain the high gravimetric density of a LiAlH_4_ vapor hydrolysis cell in line with DOE targets. Ideally, water produced via the hydrogen fuel cell reaction would be utilized to hydrolyze the lithium aluminum hydride fuel, generating hydrogen and therefore creating a constant cycle as hydrogen is then used to produce electricity and water. If additional water is required to be carried to compensate for water lost to the LDH product to ensure a continuous flow of hydrogen production, the overall gravimetric density of the system would be significantly lower than that theoretically predicted for a water-free product.

**Figure 5:**
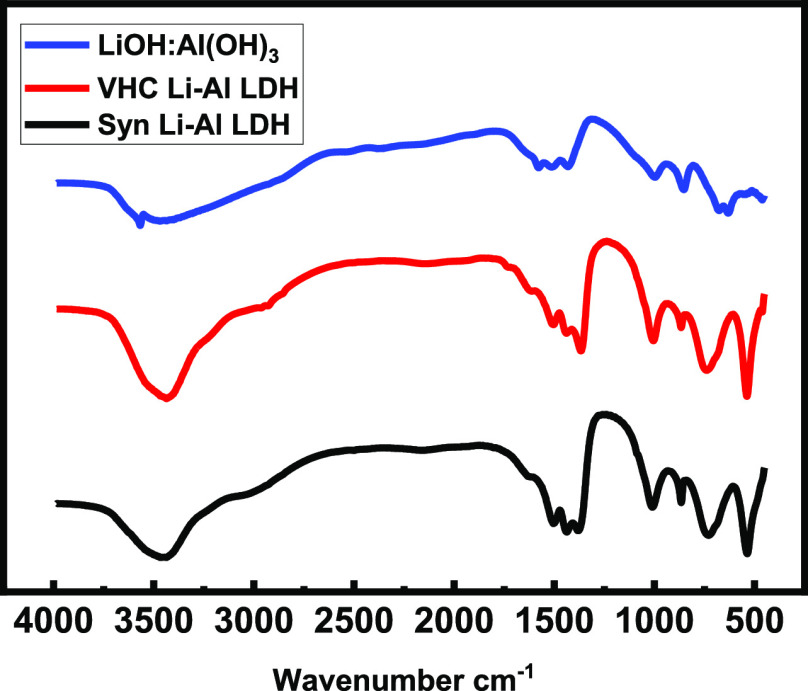
FTIR spectra of 1:1 LiOH/Al(OH)_3_ (blue), vapor hydrolysis product (red) and synthesized LDH-CO_3_ (black).

IR spectroscopy, as shown in Figure 5, further confirmed that the vapor hydrolysis of the LiAlH_4_ product, generated in the presence of carbon dioxide, differed from that of the expected simple salt products [LiOH/Al(OH)_3_].

It was again confirmed that the vapor hydrolysis product is a Li–Al double hydroxide structure, with carbonate anions in the interlayer region. Table 3 lists the assignment of IR bands. A broad transmission peak at 3450 cm^–1^ is due to structural water: water molecules present in the interlayer spacing. The bands at 1575 and 1378 cm^–1^ are attributed to the carbonate anions present in Li_2_CO_3_ and the interlayer spacing as a result of the lowering of symmetry from *D*_3h_ to *C*_2v_ and therefore splitting of ν_3_ and ν_4_. Also, at 1050 cm^–1^, the ν_1_ carbonate stretching becomes IR active. Bands at 725, 530, and 455 cm^–1^ are due to Al–O vibrations. A peak at 1030 cm^–1^ is presumed to be due to OH groups of the Al(OH)_3_ octahedral sheets, indicating the formation of the LDH structure, as reported by Chisem and Jones.^14^ A peak at 1438 cm^–1^ indicates the presence of Li_2_CO_3_.^14,18^

**Table 3:** IR Bands Assignment for the Vapor Hydrolysis Product

wavenumber cm^–1^	assignment
3450	ν(OH) H-bonding OH group in the hydroxide layer.
3000	ν(OH) H-bonding of H_2_O and CO_3_^2–^ in the interlayer spaces
1650	δ(H_2_O) water bending vibration
1575	ν_4_(CO_3_^2–^) lowered symmetry to *C*_2v_
1400	v_3_(CO_3_^2–^)
1370	v_3_(CO_3_^2–^) lowered symmetry to *C*_2v_
1050	ν_1_(CO_3_^2^)
1030	δ(H_2_O) water in the interlayer
875	ν_2_(CO_3_^2–^)
725	ν(Al–O) *A*_2u_
675	ν_4_(CO_3_^2–^)
530	ν(Al–O) *E*_u_
455	ν(Al–O) *E*_u_

**Figure 6:**
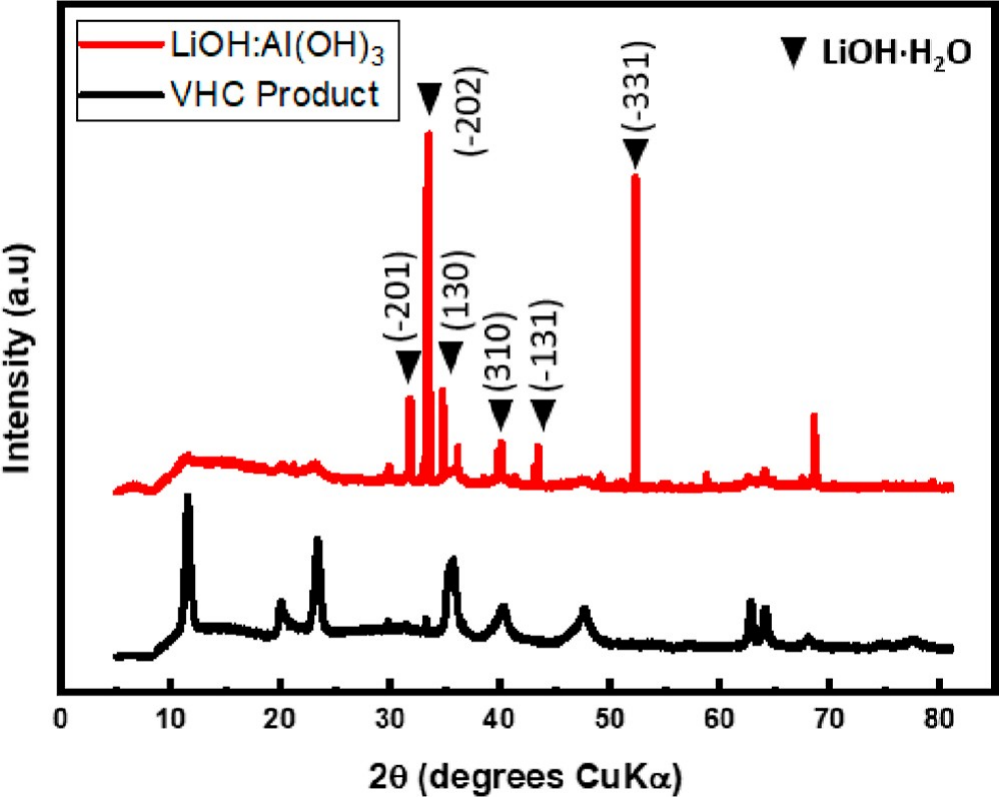
PXRD pattern of LiOH/Al(OH)_3_ (red) in a 1:1 ratio and the vapor hydrolysis product (black).

PXRD data collected for a 1:1 mixture of LiOH and Al(OH)_3_ provides further evidence that the vapor hydrolysis product is not the simple hydroxides. Figure 6 clearly shows that the actual product differs from the predicted products.

The PXRD pattern of the mixture of LiOH/Al(OH)_3_ matched that of LiOH (ICDD 25-0486).^22^ Aluminum hydroxide cannot be identified in the pattern as it is amorphous and hence no sharp reflections are observed.

### Comparison of the VHC Product with the Synthesized LDH Li–Al–CO_3_

For further confirmation that an LDH structure is formed when LiAlH_4_ is reacted with water vapor and aged for 24 h, a pre-established synthesis method for LDH-CO_3_ was followed.^14^ The characterization data of the product were compared to that of the data obtained from the VHC product.

Comparison of the IR spectrum of the synthesized [LiAl_2_(OH)_6_]_2_CO_3_·3H_2_O and the vapor hydrolysis product, produced with exposure to CO_2_, produced a spectrum match, as shown in Figure 5.

**Figure 7:**
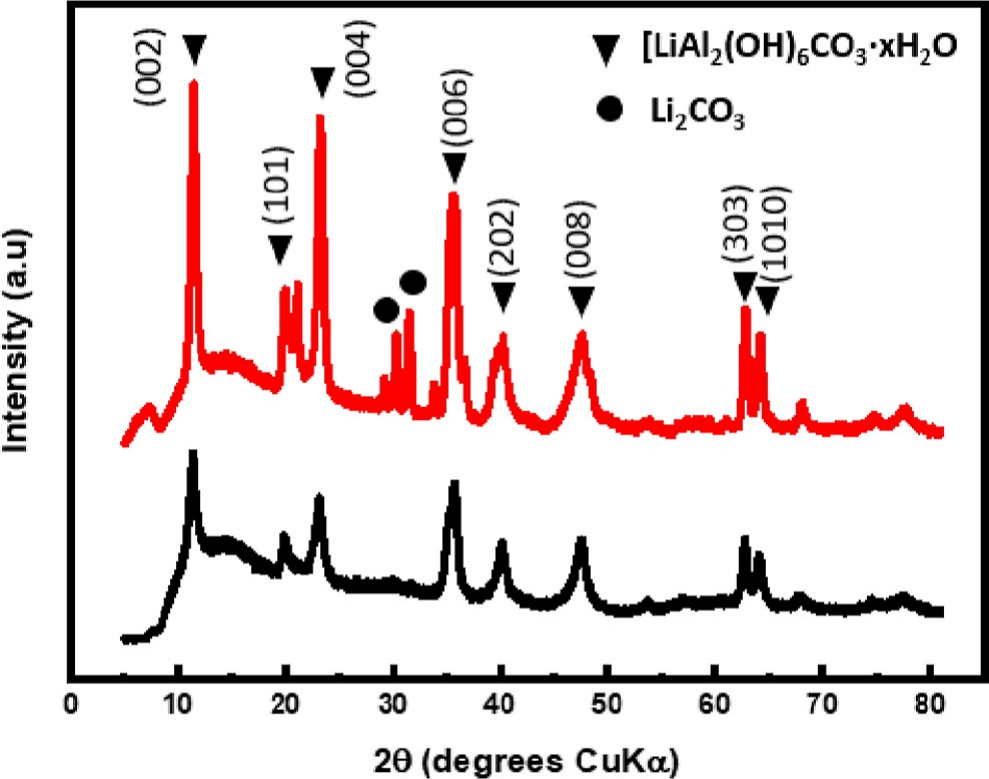
XRD pattern of the synthesized [LiAl_2_(OH)_6_]_2_CO_3_ (black) and the vapor hydrolysis product of LiAlH_4_, [LiAl_2_(OH)_6_]_2_CO_3_ production (red).

Additionally, direct comparison of the XRD pattern of the hydrolysis product with that of the as-synthesized [LiAl_2_(OH)_6_]_2_CO_3_·3H_2_O (Figure 7) further confirmed the presence of the same LDH phase. The difference in the two patterns is due to the molar ratio of 1:2 for Li/Al in the LDH product, meaning that lithium is left over from the original 1:1 Li/Al in LiAlH_4_. The excess lithium forms the simple Li_2_CO_3_ phase in the presence of CO_2_, which also appears in the pattern and is indicated by the black circles.

The XRD pattern obtained from the as-synthesized LDH-CO_3_ matched that of the vapor hydrolysis product of the LiAlH_4_ product (ICDD pattern 40-0710). Due the formation of [LiAl_2_(OH)_6_]_2_CO_3_ resulting in a lithium to aluminum atom ratio of 1:2, an additional lithium-containing compound must also be generated. As the IR spectra, TGA/DTA curves, and XRD patterns indicated the presence of Li_2_CO_3_ (ICDD pattern 09-0359),^23^ the reaction is therefore predicted to proceed via eq 4
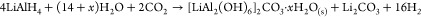
4

**Figure 8:**
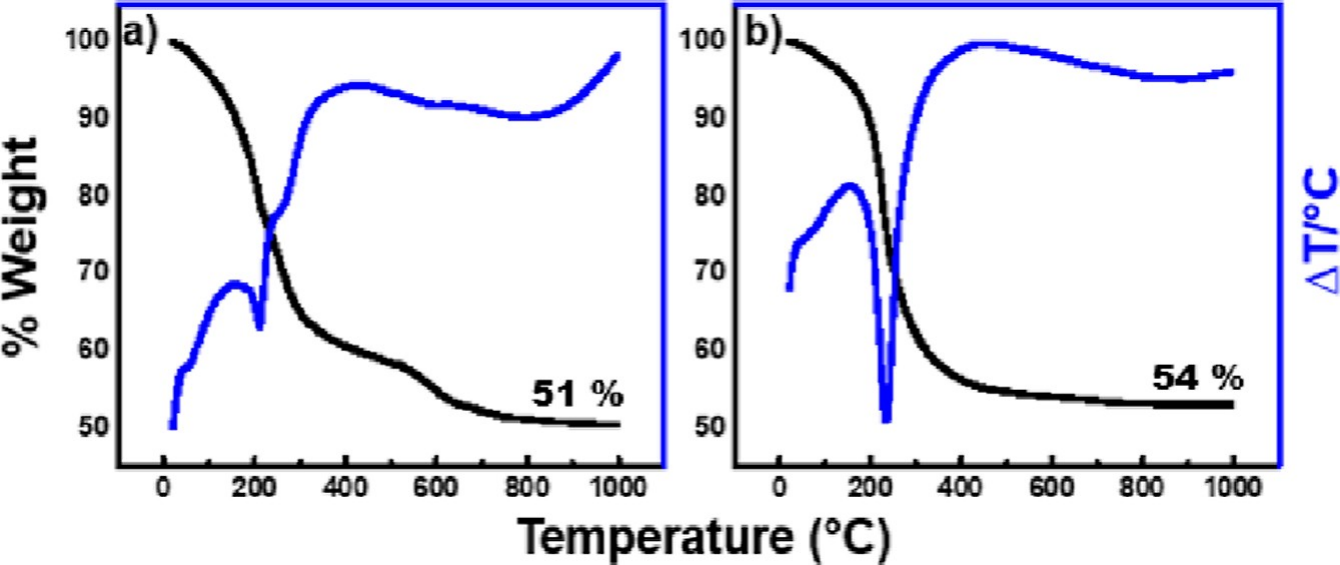
TGA and DTA data for (a) vapor hydrolysis product and (b) synthesized LDH-CO_3_.

Comparison of the TGA/DTA data from the vapor hydrolysis cell product and the synthesized LDH-CO_3_ can been seen in the figure below (Figure 8).

TGA data for the vapor hydrolysis product and the as-synthesized LDH show the continuous weight loss expected for an LDH. The percentage weight loss when heating the VHC product (a) from 25–1000 °C was 49%, while it was 46% from the as-synthesized LDH-CO_3_. This compares well with the literature value determined by Britto and Kamath of 49%.^23^

The TGA data from the synthesized LDH-CO_3_ ([LiAl_2_(OH)_6_]_2_CO_3_·*x*H_2_O) also indicates a value of *x* = 3, therefore confirming that the same LDH structure as that from the vapor hydrolysis of LiAlH_4_ is produced. An XRD analysis of the synthesized LDH-CO_3_ product after heating to 1000 °C indicates that LiAlO_2_ and Al_2_O_3_ remain. Therefore, the reactions that are predicted to occur upon heating are listed in Table 4.

**Table 4:** Reactions That Occur upon Heating of LDH-CO_3_ to 1000 °C

reaction equation	weight loss %
[LiAl_2_(OH)_6_]_2_CO_3_·3H_2_O → [LiAl_2_(OH)_6_]_2_CO_3_ + 3H_2_O	12
[LiAl_2_(OH)_6_]_2_CO_3_ → 2LiAlO_2_ + Al_2_O_3_ + CO_2_ + 6H_2_O	34

### Vapor Hydrolysis of LiAlH_4_ Performed with the Exclusion of Carbon Dioxide

Carbon dioxide contaminated the vapor hydrolysis experiment in air and hence produced a carbonate-containing LDH product. In practice, carbon dioxide will be excluded from hydrogen storage and generation systems used for fuel cells to optimize efficiency. To avoid contamination by CO_2_, vapor hydrolysis of LiAlH_4_ was performed using a glovebox and a nitrogen atmosphere. This experiment was used to determine whether or not the previously predicted products LiOH and Al(OH)_3_ would form if no carbon dioxide was present, or if an LDH structure would still be generated, but with hydroxyl anions in the interlayer spaces instead of carbonate anions.

**Figure 9:**
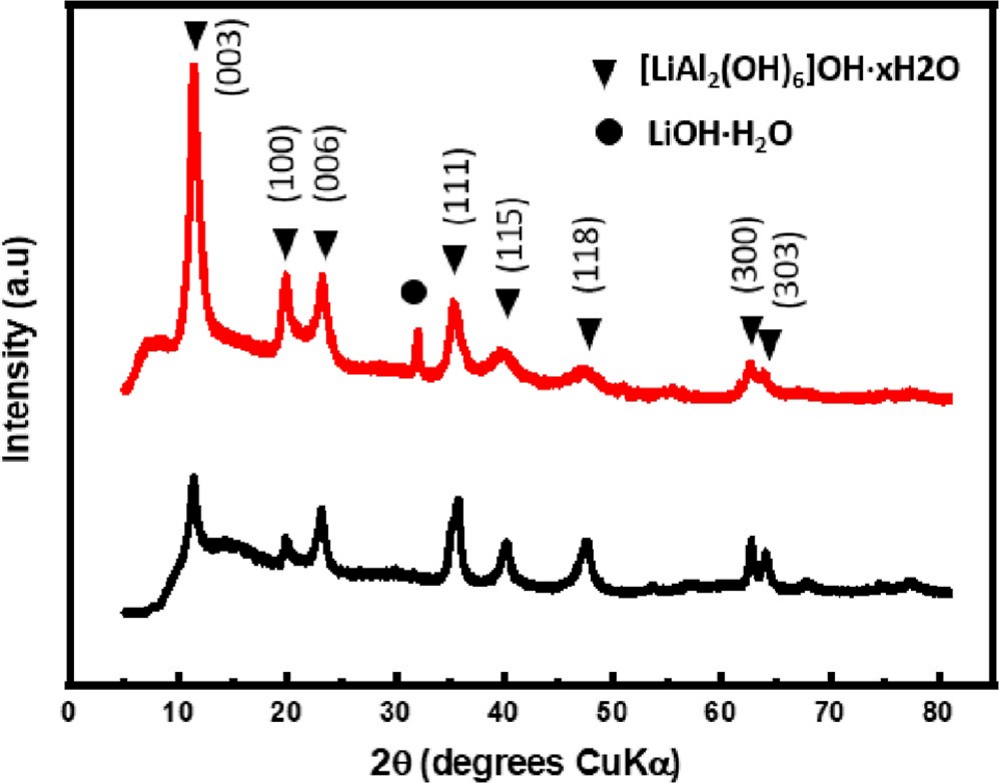
XRD pattern of the as-synthesized LDH [LiAl_2_(OH)_6_]OH (black) and the glovebox vapor hydrolysis product (red) (PDF pattern 40-0710).

The XRD pattern produced from the reaction (Figure 9) shows a match to that of the carbonate-free LDH, [LiAl_2_(OH)_6_]OH, (ICDD pattern 40-0710).^17^

Comparison of the characterization data from the glovebox-performed vapor hydrolysis to those of the product of the synthesized LDH-OH also supported that the LDH structure was formed. The PXRD pattern also indicated the presence of LiOH·H_2_O (ICDD pattern 25-0486).^23^ Therefore, the reaction is predicted to proceed as given below in eq 5
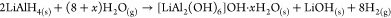
5

**Figure 10:**
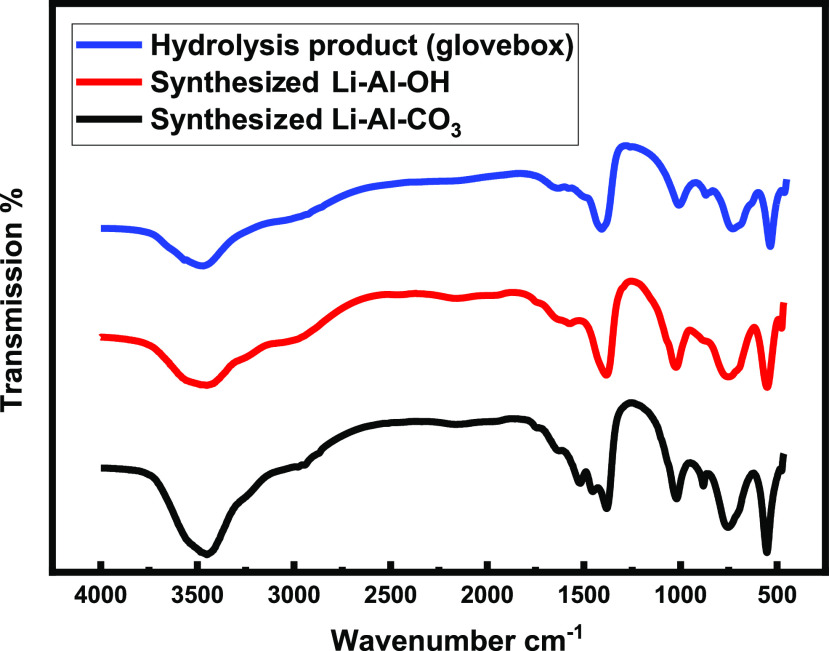
FTIR spectrum of synthesized [LiAl_2_(OH)_6_]_2_CO_3_ (red), the glovebox VHC reaction product [LiAl_2_(OH)_6_]OH (blue), and synthesized [LiAl_2_(OH)_6_]OH (black).

Figure 10 shows that the FTIR spectrum of the glovebox VHC product matches that of the synthesized Li–Al–OH as opposed to that of the synthesized Li–Al–CO_3._

There is evidence that carbon dioxide is present in the LDH-OH samples due to a peak at ∼1400 cm^–1^; however, there is an absence of the peaks associated with the lower symmetry coordinated carbonate species, which are usually only Raman active (ν_1_) or degenerate bands (ν_3_ and ν_4_); these bands are usually expected due to the coordination of the ion in the interlayer spaces of the LDH structure. Their absence suggests that predominantly water molecules and hydroxyl anions (OH^–^) are present in the interlayer spaces instead of carbonate (CO_3_^2–^).

**Figure 11:**
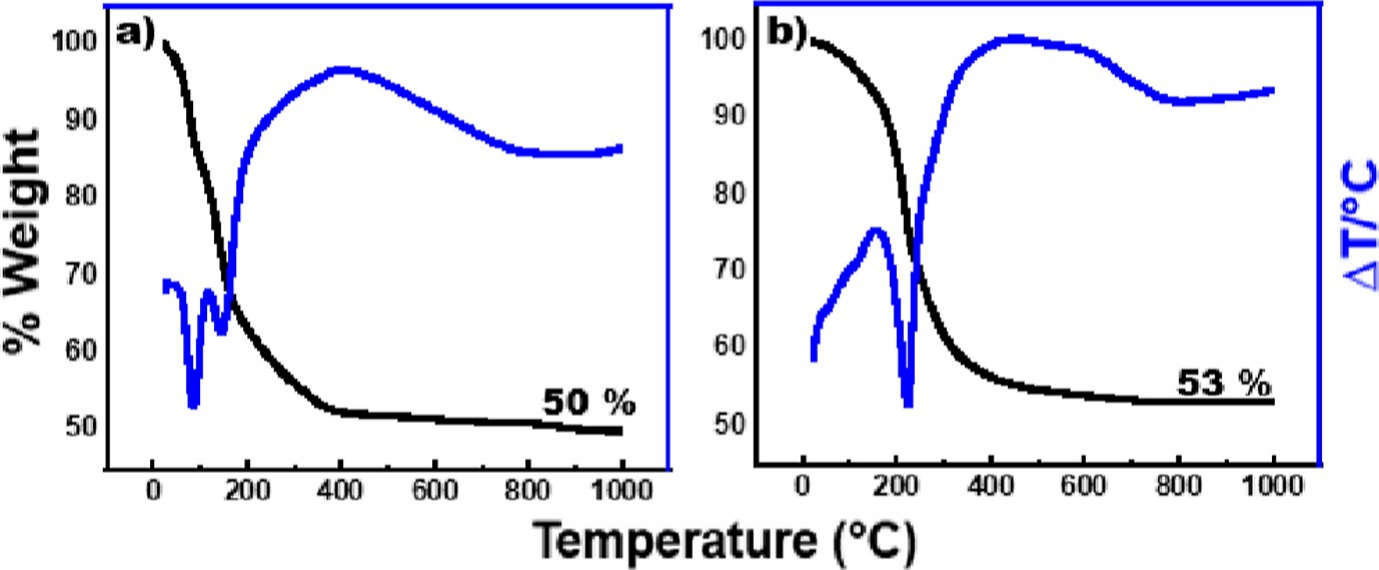
TGA and DTA data for (a) glovebox vapor hydrolysis product and (b) synthesized LDH-OH.

Furthermore, the TGA and DTA data obtained also displayed typical data expected for an LDH (Figure 11). Therefore, it can be concluded that even without the presence of carbon dioxide, an LDH product is still formed by the vapor hydrolysis of LiAlH_4_.

The products of the vapor hydrolysis of LiAlH_4_ performed with exclusion of CO_3_ were [LiAl_2_(OH)_6_]OH·*x*H_2_O and LiOH·H_2_O. After heating these products to 1000 °C, the XRD analysis of the TGA products showed LiAlO_2_. Therefore, the TGA weight loss to 50% indicates *x* = 2 as the equivalents of water of crystallization in the LDH-OH product; this agrees with the literature published by Thiel et al.^18^ The formula mass of [LiAl_2_(OH)_6_]OH·2H_2_O is 215.98 g mol^–1^; therefore, the % wt water is 17. The XRD pattern after heating the products to 1000 °C indicated that LiAlO_2_ remained. The predicted reactions that occur upon heating and the resulting weight losses are listed in Table 5.

**Table 5:** Reactions That Occur upon Heating LDH-OH and LiOH·H_2_O to 1000 °C

Reaction equation	Weight loss %
2[LiAl_2_(OH)_6_]OH·2H_2_O + 2LiOH·H_2_O → 2[LiAl_2_(OH)_6_]OH + 2LiOH + 6H_2_O	21
2[LiAl_2_(OH)_6_]OH + 2LiOH → 2LiAlO_2_ + Al_2_O_3_ + 2LiOH + 7H_2_O	25
2LiAlO_2_ + Al_2_O_3_ + 2LiOH → 4LiAlO_2_ + H_2_O	4

In comparison, the TGA data from the synthesized LDH-OH shows a weight loss to 53%, which can used to also calculate the value of *x* = 2. This therefore concludes that the LDH-OH structure is produced when performing vapor hydrolysis of LiAlH_4_ with the exclusion of CO_2_.

The XRD pattern after heating the synthesized LDH-OH to 1000 °C indicated that LiAlO_2_ and Al_2_O_3_ remained. Therefore, the reactions that are predicted to occur during the TGA are displayed in Table 6.

**Table 6:** Reactions That Occur upon Heating LDH-OH to 1000 °C

reaction equation	weight loss %
2[LiAl_2_(OH)_6_]OH·2H_2_O → 2[LiAl_2_(OH)_6_]OH + 4H_2_O	17
2[LiAl_2_(OH)_6_]OH → 2LiAlO_2_ + Al_2_O_3_ + 7H_2_O	30

### Gravimetric and Volumetric Energy Densities

When determining the energy densities of hydrogen storage materials, often, only the hydride material is included in the calculation. To give a more accurate estimation of the true energy density of a system, the other reagents must also be included.

As 1 kg of hydrogen produces ∼120 MJ (33.33 kW h), the amount of H_2_ is multiplied by this number and then divided by the mass or volume of the storage material. eq 6 is used to calculate the gravimetric energy density, while eq 7 is used to calculate the volumetric energy density.
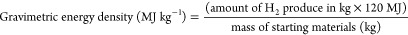
6
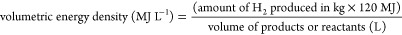
7

The previously predicted hydrolysis of LiAlH_4_ stated in eq 2 suggests that LiAlH_4_ produces 4 mol of H_2_ per mole of LiAlH_4_. As 4 mol of hydrogen gas is 0.008 kg and the total mass of starting materials is 0.11 kg, the gravimetric energy density is 8.8 MJ kg^–1^ (2.4 kW h kg^–1^).

When calculating the volumetric energy density, the largest volume must be used, either the total volume of products (not including hydrogen gas) or the total volume of reactants. The total volume of 1 mol of reactants is 0.114 L, while the total volume of products (excluding H_2_) is 0.0486 L; therefore, the volumetric density is calculated using the volume of reactants, equaling 8.5 MJ L^–1^ (2.4 kW h L^–1^). The current “ultimate” DOE targets are 2.2 kW h kg^–1^ and 1.7 kW h L^–1^; however, this must also include the overall system design.

The results demonstrated in this study highlight that instead of eq 2, an LDH structure is instead formed via eq 4, where *x* = 3. The total mass of reactants is 0.46 kg, not including carbon dioxide as this is taken from the air, and the mass of hydrogen gas produced is 0.032 kg; therefore, the gravimetric energy density is 8.5 MJ kg^–1^ (2.4 kW h kg^–1^) with respect to the reactants.

However, as the total mass of the products is 0.51 kg, the gravimetric energy density with respect to the products is 7.5 MJ kg^–1^ (2.1 kW h kg^–1^). This is a clear example of why the products should always be considered when calculating the energy density to give a more accurate estimation of the actual value, when determining if a system meets or exceeds the DOE targets.

The total volume of reactants is 0.47 L; therefore, the volumetric energy density is 8.2 MJ L^–1^ (2.3 kW h L^–1^) with respect to the reactants. As the density of the LDH product is unknown, the volume is unknown; therefore, the volumetric energy density with respect to the products cannot be calculated.

If carbon dioxide can be excluded from the reaction so that the hydrolysis of LiAlH_4_ proceeds by eq 5, where *x* = 2, the values are as follows.

The total mass of the reactants is 0.256 kg and the mass of hydrogen produced is 0.016 kg; therefore, the gravimetric energy density is 7.6 MJ kg^–1^ (2.1 kW h kg^–1^) and the volumetric energy density is 7.3 MJ L^–1^ (2.0 kW h L^–1^). Therefore, excluding carbon dioxide slightly increases the gravimetric energy density of the system but decreases the volumetric energy density.

The theoretical energy densities calculated are displayed in Table 7 as a comparison of the predicted reaction equation to the actual reaction pathway of LiAlH_4_ vapor hydrolysis.

**Table 7:** Comparison of Gravimetric and Volumetric Energy Densities of Different Reaction Pathways of LiAlH_4_ Hydrolysis

reaction equation and products.	gravimetric energy density	volumetric energy density
eq 2 LiOH/Al(OH)_3_	8.8 MJ kg^–1^ (2.4 kW h kg^–1^)	8.5 MJ L^–1^ (2.4 kW h L^–1^)
eq 3 LDH-CO_3_	7.5 MJ kg^–1^ (2.1 kW h kg^–1^)	8.2 MJ L^–1^ (2.3 kW h L^–1^)
eq 4 LDH-OH	7.6 MJ kg^–1^ (2.1 kW h kg^–1^)	7.3 MJ L^–1^ (2.0 kW h L^–1^)

The formation of LDH-OH offers a greater gravimetric energy density over the formation of LDH-CO_3_. However, the formation of LDH-CO_3_ offers a higher volumetric energy density. Therefore, excluding carbon dioxide from the hydrolysis reaction of LiAlH_4_ will maximize the volumetric energy density. This will allow extra room for system design to be incorporated; however, light materials will be required to ensure that the gravimetric energy density of the overall energy storage and delivery device meets the department of energy targets.

## Conclusions

The purpose-built vapor hydrolysis cell effectively and safely allowed the vapor hydrolysis of lithium aluminum hydride to be carried out, releasing hydrogen gas and forming an LDH product. In the presence of carbon dioxide, the LDH with carbonate anions in the interlayer spaces is formed; with the exclusion of CO_2_, an LDH with hydroxyl anions instead is produced (LDH-OH). Both products show retention of water at high temperatures, 12% wt and 17% wt for LDH-CO_2_ and LDH-OH, respectively. This is problematic for the use in hydrogen fuel cells as if water is retained by the LDH byproduct at high temperatures, then it is difficult to recycle water produced from the fuel cell reaction. Additional water will be required to be added to the system, increasing the overall weight and therefore reducing the energy density of the system. However, a gravimetric energy density of 2.1–2.3 kW h kg^–1^ and a volumetric energy density of 2.0–2.1 kW h L^–1^ can be obtained from the hydrolysis of LiAlH_4_, which exceeds the DOE targets of 2.2 kW h kg^–1^ and 1.7 kW h L^–1^, respectively. Future work should look at alternate methods of vapor hydrolysis to reduce the water retained by the LDH products formed and maximize the overall energy density of the system.
